# Social Media Use and Body Dysmorphic Disorder: A Literature Review

**DOI:** 10.1192/j.eurpsy.2026.11524

**Published:** 2026-07-31

**Authors:** S. Khalki, A. Helaoui

**Affiliations:** 1 Ibn El Jazzar Medical Faculty Of Sousse, Sousse; 2 Psychiatry Department, El Razi Hospital, Manouba, Tunisia

## Abstract

**Introduction:**

Body dysmorphic disorder (BDD) is defined as the intrusive preoccupations with perceived physical flaws, often leading to significant distress and functional impairment.

With the relatively recent rise of social media filters, photo editing tools and appearance enhancing features, it is increasingly important to study their impact on body image ideals and their potential association to BDD.

**Objectives:**

This review aims to highlight the literature findings regarding social media use and body dysmorphia among adolescents and adults.

**Methods:**

A literature review was conducted through Pubmed and GoogleScholar. The search strategy used the following terms: (“Body Dysmorphic Disorders” OR “body dysmorphia” OR “body image disturbance”) AND (“Social Media” OR “Instagram” OR “TikTok” OR “Snapchat”) A total of 67 results were identified and screened considering titles and abstracts. After excluding papers not relevant to the topic and those that were unretrievable, 34 results were included in the review.

**Results:**

Studies consistently affirmed the correlation between social media use and body dysmorphic symptoms. In fact, a study from Saudi Arabia reported a BDD prevalence of 4.2% among social media users (higher than that of the general population). Moreover, increased time spent on social media has a higher link to BDD symptoms. Particularly, appearance focused platforms, such as Instagram and Snapchat, have greater rates of negative body image than text-based content. Furthermore, women and adolescents tend to be more prone to BDD symptoms. Besides, multiple studies go even further relating social media use and BDD to other psychiatric disorders such as eating disorders and depression.

**Conclusions:**

The current literature highlights the association between body dysmorphic disorder (BDD) and social media use, but has yet to underline the mechanisms involved, which is why further research is needed. Finally, nowadays it has become important to raise awareness regarding this issue, as well as, to implement prevention and intervention strategies.

**Disclosure of Interest:**

None Declared

